# Socio-ecological determinants of lifestyle behavior of women with overweight or obesity before, during and after pregnancy: qualitative interview analysis in the Netherlands

**DOI:** 10.1186/s12884-020-2786-5

**Published:** 2020-02-12

**Authors:** Y. E. G. Timmermans, K. D. G. van de Kant, J. S. M. Krumeich, L. J. I. Zimmermann, E. Dompeling, B. W. Kramer, L. L. J. Maassen, M. A. E. Spaanderman, A. C. E. Vreugdenhil

**Affiliations:** 1 Department of Pediatrics, Maastricht University Medical Centre (MUMC+), P. Debyelaan 25, 6229 HX Maastricht, the Netherlands; 2 School for Oncology and Developmental Biology (GROW), Maastricht University, Maastricht, the Netherlands; 3 School for Public Health and Primary Health Care (CAPHRI), Maastricht University, Maastricht, the Netherlands; 4 Department of Health Ethics and Society, Maastricht University, Maastricht, the Netherlands; 5 School of Nutrition and Translational Research in Metabolism (NUTRIM), Maastricht University, P. Debyelaan 25, 6229 HX Maastricht, the Netherlands; 6 Department of Gynecology & Obstetrics, MUMC+, Maastricht, the Netherlands

**Keywords:** Lifestyle behavior, Socio-ecological model, Pregnancy complications

## Abstract

**Background:**

Maternal overweight and obesity are related to several health risks in the periods before, during and after pregnancy including a higher risk of gestational diabetes mellitus, preeclampsia and preterm birth. At the same time, women’s daily life quickly changes in these periods. Therefore, we hypothesize that the value of determinants of lifestyle behavior within different levels of the socio-ecological model differ accordingly and influence lifestyle behavior. These dynamics of determinants of lifestyle behavior in the periods before, during and after pregnancy are unexplored and therefore evaluated in this study. These insights are needed to offer appropriate guidance to improve lifestyle in women of childbearing age.

**Methods:**

Individual semi-structured interviews were conducted before, during or after pregnancy in 26 women with overweight or obesity living in the Netherlands. Questions covered all levels of the socio-ecological model, i.e. intrapersonal, interpersonal, institutional and environmental/societal. All interviews were transcribed and coded.

**Results:**

Determinants at all levels of the socio-ecological model were perceived as relevant by women of childbearing age. Various determinants were mentioned including knowledge of a healthy lifestyle, social support, access to customized lifestyle guidance, and distance to healthy lifestyle supporting activities. The importance women attributed to determinants differed between the periods before, during and after pregnancy. Before pregnancy, child’s wellbeing as motivator for adopting a healthy lifestyle was mentioned less frequently than during and after pregnancy. Women described that the interplay and balance between determinants varied on a daily basis, and not merely per period. This was often expressed as fluctuation in energy level per day which influences their willingness to put effort in making healthy choices.

**Conclusions:**

Findings of this study confirm the importance of determinants at multiple socio-ecological levels for shaping lifestyle behavior in women of childbearing age. The findings add to current insights that the perceived importance of determinants and their interplay differ before, during and after pregnancy. They influence lifestyle behavior decisions, not only per period but even on a daily basis, in particular in this phase of life. This perspective can be helpful in optimizing lifestyle guidance for women of childbearing age in order to prevent perinatal complications.

## Background

An unhealthy lifestyle in the period before, during and after pregnancy is associated with an increased time to conceive [1, 2] and a broad spectrum of health risks, such as a higher risk of miscarriage, preeclampsia, gestational diabetes mellitus and caesarean section [3–5]. Moreover, maternal obesity due to an unhealthy lifestyle is related to harmful effects on the fetus, such as a higher risk of infants born large or small for gestational age, preterm birth, and admission to the neonatal intensive care unit [6, 7]. In order to change lifestyle in women of childbearing age to improve perinatal outcomes, it is necessary to understand which determinants of lifestyle behavior are important in this period of life.

When considering determinants of lifestyle behavior, the socio-ecological model is widely used to understand interrelations between personal, social and environmental determinants [8]. The model assumes that appropriate changes in the social environment will support behavioral changes in individuals by suggesting that behavior is determined on four levels: intrapersonal, interpersonal, institutional, and environmental/societal. In a systematic review of qualitative studies among the general adult population considering primary prevention and health-promotion activities, several determinants for participation in these activities were identified within the framework of the socio-ecological model, as summarized in Table 1 [9].

**Table 1:** Determinants influencing lifestyle behavior in the different levels of the socio-ecological model

Level	Determinant	Examples
Intrapersonal	Motivation to change	Threat of disease
		Patients’ feelings of guilt and sense of responsibility
		Perception of improvement when performing prevention activities
	Knowledge and skills	Knowledge on risk factors and what is healthy
		Knowledge on sources of guidance and advice
	Self-concept	Self-esteem
		Self-confidence
	Patients’ beliefs and attitudes	Prevention is not patients’ responsibility
		Faith in the effectiveness of prevention strategies
		Fear of side effects of prevention activities
	Resources	(Lack of) financial resources
Interpersonal	Family and friends	Social and peer support
		Having a dog as a pet stimulates physical activity
Institutional	Health care professionals	(Lack of) trust in health care provider
		Judgmental approach
		Professionals’ lack of time
	System interests	Health care providers do not promote prevention activities
Environment and society	Built environment	Possibility of physical activity in the direct neighborhood, e.g. bike lanes, parks or pedestrian paths
	Cultural influence	Dietary traditions
		Social norms
		Impact of (social) media
	Socio-economic impact	(Lack of) balance between work and personal life

Determinants influencing participation in primary prevention and health promotion activities adapted from the findings of Moreno-Peral et al. [9] and summarized in this table

When supporting healthy lifestyle behavior by a lifestyle intervention it is of utmost importance to customize the intervention to the wishes and needs of the specific target population, in order to minimize barriers and maximize facilitators. Knowledge of the (value of) socio-ecological determinants of lifestyle behavior in a specific target population can lead to successful lifestyle behavior improvement [10, 11]. Thus far, lifestyle interventions with the aim to improve perinatal outcomes mainly focused on pregnant women. Although these interventions resulted in limiting gestational weight gain, disappointing effects were found on reducing perinatal complications [12]. It has been suggested that interventions earlier initiated, preferably already before pregnancy, have a higher chance on significantly reducing perinatal complications [13]. Moreover, continuation of a preconceptionally initiated lifestyle intervention during and after pregnancy might support in sustaining lifestyle improvements during a period in which women’s daily life quickly change. To be able to set up an appropriate lifestyle intervention, it is essential to have knowledge on determinants of lifestyle behavior in the specific population of women before, during and after pregnancy.

When considering previous studies on determinants of lifestyle behavior conducted in women of childbearing age, survey studies showed that women wishing to become pregnant were interested in lifestyle programs, especially when tailored to the possibilities within the daily life of women [14, 15]. During pregnancy, health of the unborn child was an important motivator for adopting a healthy lifestyle [16–18]. On the other hand, pregnant women mentioned that being pregnant is a justification for not worrying about weight and pregnancy is a time to eat for two [16]. In addition, women reported that pregnancy-related complaints and lack of time and energy due to work commitments restrain from physical activity, and that they have limited knowledge on appropriate physical activity exercises during pregnancy [16, 19–23]. In the postpartum period, lack of time and energy were often mentioned as barriers for adhering to a healthy lifestyle [24–27]. Women with a history of gestational diabetes mellitus or preeclampsia described their limited knowledge about the complication and its consequences and how to deal with that due to lack of follow-up by health care professionals after a complicated pregnancy as a barrier for lifestyle improvement [24–26].

A part of the previous qualitative research on determinants of lifestyle behavior in women during and after pregnancy was based on the socio-ecological model [20, 22, 23, 27]. The majority of these previous studies did only consider determinants of one specific lifestyle habit (for example physical activity or smoking). Exploring determinants of multiple lifestyle habits can be useful to increase insight in the complex multifactorial interplay between these habits resulting into overall lifestyle. Furthermore, in the periods before, during and after pregnancy it is hypothesized that daily life changes continuously. Previous studies did only consider determinants at a specific moment without taking into account the changes in daily life in women of childbearing age. Thereby, knowledge of determinants of making lifestyle choices in the preconception period is limited. Gaining insight in determinants of lifestyle behavior in women before, during and after pregnancy in the same study setting enables the comparison between these three life phases. Therefore, it will be possible to accentuate the specifically important determinants in each life phase. By exploring multiple level determinants, the acceptability and effectiveness of a lifestyle intervention initiated in the preconception period can be optimized. Therefore, the aim of this study was to provide a comprehensive study on determinants of multiple lifestyle behaviors within the framework of the socio-ecological model in the periods before, during and after pregnancy among women with overweight or obesity.

## Methods

### Study design, participants and setting

A qualitative study using individual interviews was performed to explore the determinants of lifestyle behavior in women of childbearing age. Women who wished to conceive within 1 year, pregnant women and women with a child younger than 1 year of age, with overweight or obesity (body mass index (BMI) ≥ 25 kg/m^2^ [28]), were included in the study. Women were excluded in case of having a hemodynamically significant heart disease, restrictive lung disease, congenital metabolic disease, diagnosis of intellectual disability according to the DSM5 criteria [29], bariatric surgery in the past, and having diabetes type II dependent on medication. Women represented all strata of society, with different educational levels, age groups, life phases and with or without previous (perinatal) health concerns. Table 2 outlines the characteristics of the participating women. Twenty-six women, aged between 24 and 36 years, participated in this study. Five women had a child younger than 1 year of age and were wishing to conceive within 1 year. Therefore, these women were interviewed about their experiences in both of these periods. The interviews took place between February and June 2015 at Maastricht University Medical Centre, the Netherlands.

**Table 2:** Participants’ characteristics

Characteristic	n (%)
BMI category	
Overweight	8 (31%)
Obesity	18 (69%)
Smoking	
Yes	4 (15%)
No	22 (85%)
Ethnicity	
Western	24 (92%)
Non-western	2 (8%)
Educational level^a^	
Low	2 (8%)
Middle	13 (50%)
High	11 (42%)
Marital status	
Not married	12 (46%)
Married	14 (54%)
Parity	
0	8 (31%)
1	14 (54%)
2	2 (8%)
3	2 (8%)
Pregnancy status	
Preconception	5 (19%)
Pregnancy	12 (46%)
< 26 weeks of gestational age	6 (23%)
≥ 26 weeks of gestational age	6 (23%)
Postpartum	4 (15%)
Postpartum & preconception^b^	5 (19%)
Pregnancy complication^c^	
Yes	3 (33%)
No	6 (66%)

All data are presented as n (% of the whole group of participants)

^a^Educational level was divided in low (completed primary school), middle (completed secondary school or secondary vocational education) and high (completed higher professional education or university)

^b^This group includes women who had a child younger than 1 year of age and were trying to conceive again

^c^These numbers and percentages are related to the women of the postpartum group

### Interviews

Data were collected by using individual semi-structured interviews. Each interview was conducted by either YT or LM, two well-trained researchers who were unknown to the participants prior to the interviews. The first four interviews were conducted in the presence of the second researcher in order to reach agreement about the communication style, and the manner to approach the women during the interviews. Repeat interviews were not carried out. The mean duration of the interviews was 42 min and 11 s (standard deviation 13 min and 37 s). Audio recordings from the interviews, with permission of the interviewees, were used to collect data. The interview guide was developed and based on the socio-ecological model. The interview guide consisted of questions on current experiences regarding different aspects of lifestyle; smoking, nutrition and physical activity. In addition, potential barriers and facilitators of smoking cessation, and nutrition and physical activity according to the recommendations of the Netherlands Nutrition Centre and the Health Council of the Netherlands [30, 31] were asked. Questions in the interview guide covered all levels of the socio-ecological model. The full interview guide is presented in Additional file 1. At the moment no new insights could be extracted from the interviews and data saturation was reached participant inclusion stopped. This method was applied on two levels: 1) insights in determinants for lifestyle behavior that were relevant for the entire target group; and 2) for each subgroup; women before, during and after pregnancy.

### Recruitment and ethical concerns

Women who wished to conceive within 1 year, pregnant women and women with a child younger than 1 year of age, with overweight and obesity were the most suitable participants to achieve the primary aim of this study [32]. Therefore, purposive sampling was used to specifically reach this target group. Subjects were recruited via midwifes, gynecologists or via advertisements in the lay press and social media. After women agreed to be approached by the researchers, the researchers discussed the aim and design of the study with the women by telephone or face-to-face. Twelve women declined to participate or did not meet inclusion criteria. Each woman that agreed to participate, was included in consecutive order. The researchers reinforced that it was not possible to give incorrect answers during the interviews. During the interviews, the researchers devoted particular attention on a sympathetic approach of the women, and on not being judgmental. This qualitative study was approved by the ethics committee of the Maastricht UMC+ (METC 14–4-159) and all participants signed informed consent before the interviews took place.

### Data analysis

Directive content analysis as described by Hsieh and Shannon [33] based on the socio-ecological model was applied for data analysis. The socio-ecological model and the four levels that are identified within the framework of this model were the basis for the interview guide. After interviews were performed, audio recordings were converted into transcripts, and participants’ names were coded. Transcripts were reviewed carefully, and all text was highlighted that appeared to describe a determinant of lifestyle behavior. All highlighted text was coded using the four levels of the socio-ecological model wherever possible. Highlighted text that could not be coded into one of these categories was coded with another code that matches with the content of the highlighted text. Subcategories within the four levels of the socio-ecological model were defined. In addition, determinants of lifestyle behavior that were relevant for a specific period (before, during or after pregnancy) were categorized separately. Interviews were independently coded by both investigators to control for inter-observer variation. Differences regarding codes were discussed by the two investigators until consensus was reached.

Five women were wishing to conceive within 1 year (preconception) and had a child younger than 1 year of age (postpartum). Specific themes in the preconception or postpartum period that arose from the interviews with these women were assigned to the appropriate period. The interviews were transcribed in NVivo (NVivo qualitative data analysis software; QRS International Pty Ltd. Australia; Victoria Version 12). In addition, NVivo was used to code and to organize the data derived from the interviews. Transcripts and findings were not returned to participants. Both researchers kept a self-reflective diary to evaluate their own subjective views on the interpretation of the interviews. In this study, we adhered to the COnsolidated criteria for REporting Qualitative research (COREQ) guidelines [34].

## Results

Analysis of the interviews showed three main themes that affect lifestyle behavior in the participants, which will be further elaborated: 1) determinants within the framework of the socio-ecological model; 2) specific determinants within the preconception, pregnancy and postpartum periods; 3) the dynamic nature and context in which people make choices.

### Determinants within the framework of the socio-ecological model

In general, the interview data showed different determinants of lifestyle behavior in the target group within each level of the socio-ecological model. The determinants that are highest valued by the participants are summarized in Table 3. Several examples are further explored below. The term “lifestyle” is defined as a combination of multiple lifestyle behaviors including nutrition, physical activity and smoking habits.

**Table 3:** Description of determinants of lifestyle behavior within the framework of the socio-ecological model

Level	Determinant	Examples
Intrapersonal	Physical and mental wellbeing	Physical complaints interfere with the willingness for physical activity
		Psychological problems need to be solved first
	Knowledge	Having knowledge of a healthy lifestyle
		Having knowledge of resources for guidance in lifestyle improvement
	Attitudes	By adopting a healthy nutrition pattern, it is hard to enjoy social activities such as a dinner
		Unhealthy food and smoking are an addiction and are therefore difficult to change
		Do not like exercising or cooking
		Having anxiety for physical activity
	Motivation to change	A healthy lifestyle supports the improvement of wellbeing, physical fitness, health
		A healthy lifestyle stimulates a positive body image
	Self-confidence	It is difficult to resist temptations regarding food
		It is difficult to persevere lifestyle changes
Interpersonal	Social environment	Receiving compliments about lifestyle changes stimulates to persevere
		Support from social environment in terms of changing lifestyle together and helping in making healthy choices stimulates to initiate and persevere lifestyle changes
Institutional	Group sessions	Group sessions stimulates bonding with other women in the same situation
		Group sessions create social pressure for participating
	Provision of information	Contradictory provision of information creates confusion about what is healthy
	Health care professional	Women prefer to have one contact person for each participant and relationship of trust
		Monitoring and positive reinforcement stimulates to persevere lifestyle changes
	Content of intervention	Women prefer intervention content to be of multiple disciplines
		Women prefer pragmatic and achievable advices, no extreme regimens
		Every woman feels unique and preferred to be treated accordingly
Environment and society	Work/life balance	Irregular working hours are a barrier for having a balanced nutrition pattern and for routine physical activity
		Physically active work stimulates being physically active
	Neighborhood	Activities organized within lifestyle intervention in direct neighborhood stimulates participation, especially regarding weekly physical activities
		Bike lanes, urban environment and pedestrian paths stimulate physical activity
	Public policy	Compensation by insurance company stimulates participation in lifestyle guidance
		Discounts for healthy products such as healthy food or sport subscription stimulate buying these products and making healthy choices

On intrapersonal level, participants experienced and believed that their wellbeing, physical fitness, body image and health could be improved by a healthy lifestyle. In turn, women mentioned that these convictions increased their motivation to adopt a healthy lifestyle.

*Participant 23 (25–29 years of age; parity 1; postpartum): “[…]and at that moment, you notice that your weight is hindering. And that you are affected by the extra body weight and kilograms when being physically active and that your physical capacity has decreased.”*

Also, some women noted that they would like to increase their knowledge of components of a healthy lifestyle in order to be able to improve their lifestyle. The search for clear, unequivocal information supporting a healthy lifestyle was experienced to be difficult. This was assigned to the large body of contradicting advice on the Internet, in books or in magazines. Therefore, women indicated that there is a demanding need for reliable information.

*Participant 1 (30–34 years of age; parity 0; pregnant): “There are a lot of diet books available, but, one book recommends to do this and another book recommends to do it like that, […]what is actually the healthy choice?”*

On interpersonal level, almost all women felt the desire to receive social support from their loved ones in adopting a healthy lifestyle. In addition, some women preferred group-based interventions, to build new relationships with women being in the same situation, and to encourage each other to continue participation in prevention activities.

*Participant 5 (30–34 years of age; parity 0; pregnant): “My husband is working on a healthy lifestyle as well […]. Consequently, he motivates me to go exercise with him and to pay attention on making healthy choices.”*

In terms of institutional determinants, a clear preference for a multidisciplinary program was noted, with one contact person per participant to ensure a relationship of confidence. Furthermore, women noted that every woman is unique with a different work/life balance, other personality, other preferences, and other motivation. Therefore, women indicated that lifestyle coaching should be customized to the needs and possibilities of the woman.

*Participant 9 (30–34 years of age; parity 1; pregnant): “Off course, it is so personal, every person is different […]. It is possible to start a running club but when five out of ten people do not like running, sooner or later they will drop out.”*

When considering the influence of the environment and society, main themes that were mentioned were the built environment, cultural context and employment conditions. The natural and urban environments in the direct neighborhood were indicated to play a role in stimulating physical activity. On the other hand, irregular or fulltime working hours refrained women from sustaining a healthy nutrition pattern or being physically active on a frequent basis while physically intense work increased physical activity.

*Participant 8 (30–34 years of age; parity 0; pregnant): “My job is rather demanding, not physically, but mentally, taking decisions. And I notice that, yeah, that I work fast. And that demands my physical and mental capacities, so in the evening I do not have the energy anymore to go out for a workout.”*

### Specific determinants within the preconception, pregnancy and postpartum periods

In addition to the determinants in the socio-ecological model as described in the previous paragraph, women mentioned determinants of lifestyle improvement that are specifically relevant in the periods before, during and after pregnancy. Before pregnancy, women perceived the determinants in the socio-ecological model which are also applicable to the general population as most important to influence their lifestyle behavior. In particular in the preconception period, some women mentioned that weight gain is a consequence of hormone treatment as part of the fertility treatment, which interferes with the motivation to make healthy choices.

*Participant 3 (35–39 years of age; parity 0; preconception): “With every hormone injection I gain 5 kg of body weight.”*

However, on intrapersonal level women were more motivated to adopt a healthy lifestyle before pregnancy because of their believe that it becomes easier to conceive, and to prevent pregnancy complications and excess gestational weight gain.

*Participant 24 (35–39 years of age; parity 1; preconception): “Once I have read that when you are overweight, that becoming pregnant could take a longer time […] I would like to prevent that.”*

*Participant 18 (20–24 years of age; parity 1; postpartum and preconception): “Actually I only want to become pregnant when my weight is adequate. Just … that is the best for the baby in my belly, the risk on gestational diabetes is high.”*

During pregnancy, pregnancy-related complaints such as pelvic girdle pain, nausea and having less energy, and the increased belly size itself were found to be obstacles on intrapersonal level for being physically active or eating a healthy diet. In addition, the women’s sense of taste was affected by pregnancy resulting in a preference for more healthy or unhealthy food. Several women felt a lack of energy during pregnancy and consumed extra calories for compensation. In addition, some women used pregnancy as a justification for postponing changing their nutrition pattern or start physical activities. When considering institutional determinants during pregnancy, knowledge about safe food enabled women to make well-informed food choices during pregnancy.

*Participant 4 (25–29 years of age; parity 1; pregnant): “During the current pregnancy I was nauseous during the first four months. Consequently, I did not eat that much.”*

*Participant 5 (30–34 years of age; parity 0; pregnant): “Currently, I often feel the need for more nutritious food than only a salad, so I try to alternate between a salad, toast or a bread roll.”*

*Participant 4 (25–29 years of age; parity 1; pregnant): “Of course, I thought about changing my lifestyle. But I do not think I will … I think it is also possible after pregnancy. Haha.”*

After pregnancy, women indicated on interpersonal level they were repeatedly seeking for a new daily rhythm including the sleep-feed rhythm of the child and the care of other family members which makes it difficult to adhere to healthy food patterns and sustain in physical activities. In addition, determinants on intrapersonal level such as recovery from the delivery or mastitis were determinants that played a role in changing lifestyle. On the other hand, women said they would like to set a good example for their child and would like to be able to be physically active together with their child in order to stimulate their child’s motor skill development. On environmental level, limited availability of a baby-sitter (either limited financial sources or limited social contacts) was also perceived as barrier for performing physical activities.

*Participant 7 (35–39 years of age; parity 1; postpartum and preconception): “My daughter eats quite a lot already. So I would like that she learns what healthy food is, no matter how young she is.”*

*Participant 15 (30–34 years of age; parity 1; postpartum): “Well, after having mastitis for the third time you have to choose for yourself and stop breastfeeding. Because, well, for a couple of days I was really suffering.”*

*Participant 12 (35–39 years of age; parity 1; pregnant): “After my pregnancy, I started with lifestyle guidance again and it took a period of time to lose weight compared to the time when we did not have any children. […] I was tired more quickly and, more easily chose food items that were not healthy.”*

#### Differences in perceived determinants in the periods before, during and after pregnancy

In the different periods before, during and after pregnancy, women valued determinants alternately as described in the previous paragraph. By comparing the different periods, a distinct difference was noticed in the threat women felt about the wellbeing and health of their child between women in the different periods before, during and after pregnancy. In general, women mentioned that the wellbeing and health of their child was the most important stimulator for adhering to healthy lifestyle guidelines. This phenomenon was clearly more evident in the periods during and after pregnancy than in the preconception period.

*Participant 6 (20–24 years of age; parity 0; pregnant): “Now I am pregnant, I try to live healthier, because […] there is living something in me. Consequently, we are more concerned about our food choices.”*

*Participant 18 (20–24 years of age; parity 1; postpartum and preconception): “I read an article about overweight in parents. When parents are overweight, children are at higher risk to become overweight. Well, I know the consequences of being overweight, and I do not want my daughter to experience the same.”*

### The dynamic nature of making choices over time

Besides the various determinants that influence lifestyle behavior as mentioned in the previous paragraphs, women specifically mentioned that the importance they attributed to these determinants changes every day. On daily basis, women experienced fluctuating levels of energy, distress, and positive or negative emotional feelings. The motivation to adopt to a healthy lifestyle because women believe that this will support their own and their child’s wellbeing and health can be overruled by these fluctuating feelings. For instance, when women have a low energy level, the motivation to adhere to a healthy lifestyle can be overshadowed by the fact that the women have no energy to be physically active. In addition, the intention to make healthy lifestyle choices can be influenced by spontaneous or unexpected social activities such as a dinner or a birthday party.

*Participant 26 (30–34 years of age; parity 1; postpartum and preconception): “I established such a strict schedule for eating, but it is difficult when I am at a different place with friends or family, or when we visit friends or family. What should I do at those moments?”*

*Participant 5 (30–34 years of age; parity 0; pregnant): “It depends on what clothes I wear that day, what my feelings are, and what day of the week it is. One day I think: I am elegantly. Another day I think: I really need to do something about my overweight.”*

*Participant 25 (30–34 years of age; parity 0; preconception): “Of course, there are some days that I am apathic, when I come at home I lay down on the couch. […] And then I am just staring […] or watch television while at that moment I would have had time for physical activity.”*

*Participant 16 (25–29 years of age; parity 3; postpartum): “Sometimes, there are periods in which I feel sick and insecure about myself. Those are things, that slow me down in adopting a healthy lifestyle. I really need to encourage myself at those moments.”*

## Discussion

In this study, several determinants at all levels of the socio-ecological model were found to influence lifestyle behavior in women of childbearing age. Determinants mentioned as being important by the women included: the believe that a healthy lifestyle supports wellbeing, physical fitness and health, knowledge about a healthy lifestyle, social support, access to personal and customized lifestyle guidance, and the distance to lifestyle guidance activities. In addition, specific determinants of lifestyle behavior in the periods before, during and after pregnancy were found. Before pregnancy, important determinants of lifestyle behavior were mainly equal to the determinants applicable to the general population. The most important motivation to adopt to a healthy lifestyle mentioned by the women in the pregnancy and postpartum period was to support their child’s wellbeing and health. Physical complaints during pregnancy and continuously seeking for a daily rhythm with their new born in the postpartum period were mentioned as barriers for a healthy lifestyle. Besides, we found a dynamic interplay and difference between the periods before, during and after pregnancy in the importance of determinants perceived by the women that affected lifestyle behavior decisions.

The determinants in women of childbearing age as found in this study, were comparable to determinants of lifestyle behavior in the general population as found in previous studies [9, 16, 19, 35–38]. The findings of period specific determinants are especially relevant when supporting women to adopt a healthy lifestyle continuously in the periods before, during and after pregnancy. The determinants of lifestyle behavior in the pregnancy and postpartum periods are in line with the findings described before [16–24, 39]. The current research is an important addition to the existing knowledge in identifying preconception period specific determinants. In addition, the finding that women in the preconception period felt the threat about the wellbeing and health of their child less compared to women in the pregnancy and postpartum period adds to the current knowledge. Furthermore, previous studies might be at risk for recall bias because of their retrospective design [18, 20]. In the current study, women were interviewed about their current experiences with the purpose to prevent recall bias. Moreover, in previous studies, the importance of determinants of lifestyle behavior were often presented as being rigid. In the current study, women described that they experience every day as different. This indicates that women are striving every day for a balance between their motivation and the barriers and facilitators they encounter. One day the motivation to make healthy lifestyle choices might be overshadowed by barriers while the other day the motivational determinants and facilitators predominate. So far, most studies did not report on this dynamic nature of people making choices and the changing context in which they are making choices as found in this study. However, this phenomenon was earlier described in the context of using preconception care by couples planning pregnancy [40]. The authors concluded that deciding on using preconception care is a subtle process in which women might shift their perceptions depending on time and context. This suggests that the determinants of lifestyle behavior and the interplay between them are not that fixed or rigid as earlier described, but are dependent on the moment and place in which people make lifestyle behavior decisions. Especially in women of childbearing age who are going through a period in their life that is subject to many changes in daily life, it is important to take this phenomenon into consideration when supporting them in making healthy lifestyle decisions.

Women in the current study mentioned that they were motivated to adopt a healthy lifestyle because they previously experienced and believed that it will improve their wellbeing, physical fitness and health. As found in this study, and based on previous research, the wellbeing and health of the (unborn) child contributed even more to the motivation for making healthy lifestyle choices [16–19]. The phenomenon that people’s motivation for lifestyle change is dependent on the perceived health risk, was also reflected by studies that involve women with (a history of) a mental, physical or reproductive health problem, or a previous miscarriage. These women were more aware of health risks and had a higher need for preconception-related information [41–43]. In the current study, women in the period before pregnancy were less aware of risks for the wellbeing and health of their (unborn) child than women during and after pregnancy. Therefore, women might be less likely to recognize the importance for healthy lifestyle behavior in the period before pregnancy [40, 44].

Findings of this study might have some important implications for the public health domain since determinants for lifestyle improvement specifically relevant for women of childbearing age were observed. Taking into account these determinants will facilitate the optimization of the acceptability and the effectiveness of the lifestyle intervention on prevention of perinatal outcomes. It is known that almost all couples planning a pregnancy have one or more lifestyle-related risk factors for developing perinatal complications [45]. However, women in the preconception period are less aware of these lifestyle-related health risks. This gap between perceived and actual health risk might be narrowed by educational campaigns. Studies evaluating the effects of educational campaigns have shown that these were associated with improvement of preconception lifestyle and that they have the potential to increase the use of preconception care [46, 47]. However, it was demonstrated that these campaigns do not reach the entire population. Especially women of lower educational levels were not easily reached [46]. It is therefore suggested that increased awareness of health risks and lifestyle behavior improvement requires a mix of interventions [48]. In any case, when women participate in a lifestyle intervention, information on health risks for both mother and child should be provided. Preferably, this information is individualized to the risk factors applicable to a particular woman. Second, women felt to be unique with their own motivations and activities in their daily life and emphasized that they would like to be treated in that way. Therefore, lifestyle guidance should be customized to the woman’s needs, wishes, and possibilities within her daily life which is in line with recommendations from previous studies [17, 18]. This can be facilitated by offering a broad range of lifestyle supporting programs. In that way, woman can assemble the combination of supporting programs that matches their needs the best. As suggested in previous reports [14, 15], a lifestyle intervention in which health care providers are flexible in their planning and location might be beneficial in increasing women’s motivation to participate. Third, a lifestyle intervention should consider the daily dynamics of women making their choices dependent on their feelings and (emotional) wellbeing of that day. Anticipating on possible unpleasant moments or days and how to deal with that, can women help to cope with these moments and make healthy lifestyle choices.

It is widely accepted that findings of studies are not universally transferable, as is also the case for the current study [49]. Although the participants included in this study were heterogenous with regard to age, social status, parity and marital status, all women were living in the South of Limburg (the Netherlands), all women had overweight or obesity and the minority of women had a low educational level. Therefore, caution is needed when generalizing these findings and their implications to other populations of childbearing age. On the other hand, including women all living in the South of Limburg and in the same time period in this study made it possible to evaluate the differences in determinants of lifestyle behavior in the life phases before, during and after pregnancy with each other. Furthermore, part of our findings was comparable to the findings of other studies conducted in other countries and among people with different weight categories. This indicates that at least these findings are important to take into account for optimizing a lifestyle intervention.

## Conclusions

Determinants at each level of the socio-ecological model and period specific determinants were found to influence lifestyle choices by women before, during and after pregnancy. Important determinants to take into account included knowledge on a healthy lifestyle, social support, access to personal and customized lifestyle guidance, and healthy lifestyle supporting activities in the direct neighborhood. Also, physical complaints during pregnancy and continuously seeking for a daily rhythm with their new born in the postpartum period were found to be important determinants. In addition, day-by-day fluctuations in the importance and interactions between these determinants were observed. Considering that the balance between the importance of determinants over time influences lifestyle behavior will be helpful in optimizing lifestyle guidance for women before, during and after pregnancy in order to prevent perinatal complications.

## Supplementary information

**Additional file 1.** Interview guide.

## Data Availability

The datasets generated and/or analyzed during the current study are not publicly available due to the ethical concerns related to the identifying personal information but are available from the corresponding author on reasonable request.

## Abbreviations

BMI: body mass index
COREQ: COnsolidated criteria for REporting Qualitative research
