# Supplementary material for: Socio-ecological determinants of lifestyle behavior of women with overweight or obesity before, during and after pregnancy: qualitative interview analysis in the Netherlands
Source: BMC Pregnancy Childbirth. 2020 Feb 12;20:105. doi: 10.1186/s12884-020-2786-5 (PMC7017483; doi:10.1186/s12884-020-2786-5)
Supplement: Supplementary file 1 — Additional file 1. Interview guide. [file 12884_2020_2786_MOESM1_ESM.docx]

**Additional file 1: Interview guide**

**Demographics**

| Have you been pregnant before?   - How many weeks are you pregnant at this moment? - Did you encounter any complications during pregnancy or delivery? |
| --- |
| How many children do you have? |
| Are you married? |
| Do you smoke at this moment? If not, have you ever smoked, and when did you quit smoking?   - At what age did you start smoking? - Does your partner smoke? |
| What is your birth date? |
| What is your height and weight?   - What was your preconception weight? |
| What is your highest educational degree? |
| Are you currently employed?   - What is your present / last profession? |
| What is your nationality? |

Try to achieve in-depth answers. Example questions to ask open-ended supplementary questions:

- Can you explain that?
- What do you mean by that?
- Can you give an example?

**Daily life**

| Can you describe your daily life?   - What kind of activities are incorporated in your daily life? |
| --- |
| Are there any relaxation activities incorporated in your daily life? |
| Can you elaborate on your diet?   - Ask supplementary questions when needed. |
| Can you elaborate on your physical activities?   - Ask supplementary questions when needed. |
| Under what circumstances did you start smoking? |
| Did you ever participate in a lifestyle program? |

**Health**

| What does “health” mean to you? |
| --- |
| Do you search for information about your health?   - Ask supplementary questions on search for information regarding nutrition, physical activity and smoking. |
| Are you aware of/affected by the health of your (unborn) child and how do you express this?   - Ask supplementary questions about nutrition, physical activity and smoking. |

**Change in daily life**

| Are there aspects of your daily life changed now you are planning to conceive/are pregnant/have a child younger than one year of age and what has been changed?   - Ask supplementary questions on nutrition, physical activity and smoking. - Do you participate in any specific activities directed to your pregnancy such as childbirth courses or sport activities for pregnant women? |
| --- |
| Are you satisfied with your daily life at this moment?   - Are there aspects in your daily life/lifestyle that you would like to change? - What are reasons for you to (not) make any changes in your daily life/lifestyle? - Are there any barriers that you experience when you would like to change aspects in your daily life/lifestyle? - Are there any facilitators that would help/support you in changing aspects in your daily life/lifestyle? - Keep in mind the three lifestyle behaviors nutrition, physical activity and smoking when considering the above-mentioned questions. |
| In the past, did you ever make changes to your diet, physical activity and/or smoking status?   - Were there any activities/support that helped you with changing these behaviors? - Were there any barriers that you encountered when trying to change these behaviors? |
| What is your opinion about taking medicines as support for smoking cessation? |

**Social support**

| Do you notice that your social environment has an opinion about your lifestyle? |
| --- |
| What does the opinion from your social environment mean for you? |
| Do you experience any support from your social environment? Can you explain this? |
| Do you feel any social pressure regarding your lifestyle behavior? Can you explain this? |

**Built environment**

| The physical activities that are incorporated in your daily life, are there any outside activities included? |
| --- |
| Is it easy to walk or cycle in the neighborhood of your home? |
| Are there any other barriers or facilitators that you encounter in the neighborhood of your home? |

**Lifestyle guidance**

| Let’s imagine that you will participate in a lifestyle guidance program, can you describe how an ideal program will look like? Examples of items that you might consider in a lifestyle intervention, are:   - Provision of information. - What kind of activities? - What kind of contact moments (face-to-face, by telephone, by e-mail)? - What kind of health professionals? - Results that the program needs to bring for you. - Social support in the program, would you need that? - Practical issues such as the location of activities/consultations, time window of activities/consultations, time investment, any costs involved in the lifestyle guidance program, group or individual sessions. |
| --- |
| Are there any barriers that you would experience when participating in such a lifestyle guidance program? |
| Are there any stimulating factors/facilitators that you would experience when participating in such a lifestyle guidance program? |
